# Sex differences in exercise‐induced arterial hypoxemia and pulmonary edema following high‐intensity exercise in highly trained endurance athletes

**DOI:** 10.14814/phy2.70190

**Published:** 2025-01-09

**Authors:** Alanna S. Hind, Reid A. Mitchell, Olivia N. Ferguson, Morgan Flynn, Satvir S. Dhillon, Karine Badra, Kathryn M. Milne, Danilo Iannetta, Michael S. Koehle, Jordan A. Guenette

**Affiliations:** ^1^ Centre for Heart Lung Innovation The University of British Columbia (UBC) and St. Paul's Hospital (SPH) Vancouver British Columbia Canada; ^2^ Department of Physical Therapy UBC Vancouver British Columbia Canada; ^3^ Department of Emergency Medicine SPH Vancouver British Columbia Canada; ^4^ Division of Respiratory Medicine UBC Vancouver British Columbia Canada; ^5^ Department of Anesthesiology, School of Medicine University of Utah Salt Lake City Utah USA; ^6^ School of Kinesiology UBC Vancouver British Columbia Canada; ^7^ Division of Sports Medicine, Department of Family Practice UBC Vancouver British Columbia Canada

**Keywords:** arterial hypoxemia, b‐lines, exercise physiology, pulmonary edema, respiratory physiology, ultrasound

## Abstract

This study investigated sex differences in the development of pulmonary edema and exercise‐induced arterial hypoxemia (EIAH) in well‐trained endurance athletes during near‐maximal exercise in a real‐world setting. Twenty participants (10M vs. 10F; V̇O_2_peak: 69.3 (8.8) vs. 50.7 (4.1) ml∙kg^−1^∙min^−1^) underwent a maximal incremental treadmill test (visit 1) and a time trial on a steep trail (~2.5 km, ~800 m elevation gain) in North Vancouver (visit 2). Pulmonary edema was evaluated using handheld lung ultrasound ~10–15 min post‐exercise and oxygen saturation (SpO_2_) was monitored using finger pulse oximetry. Males completed the time trial significantly faster than females (M: 31.5 (6.5) vs. F: 40.4 (7.5) min, *p* = 0.006), while females sustained a higher percentage of their visit 1 heart rate (M: 94 (1) vs. F: 96 (1) %max, *p* = 0.02). All participants developed EIAH, with no sex differences in end‐exercise SpO_2_ (M: 89 (4) % vs. F: 90 (3) %, respectively, *p* = 0.35). There was no evidence of pulmonary edema, assessed through ultrasound b‐line scores, with no differences between sexes (M: 0.3 (1.0) vs. F: 0.5 (1.5), respectively, *p* = 0.60). Pulmonary edema is an unlikely contributor to EIAH in endurance athletes performing near‐maximal time trial exercise in a real‐world setting.

## 1 INTRODUCTION

It is well established that females have smaller lungs, narrower airways, and a reduced surface area for pulmonary gas exchange relative to height‐matched males (Dominelli et al., 2018; Mead, 1980; Sheel & Guenette, 2008). These differences may contribute to increased pulmonary limitations in females during exercise (Dominelli et al., 2019). Notably, research suggests that females may be more susceptible to exercise‐induced arterial hypoxemia (EIAH) than males (Dominelli & Molgat‐Seon, 2022; Richards et al., 2004); although this is not a universal finding (Hopkins et al., 2000). EIAH is defined as a reduction in arterial oxygen saturation (SaO_2_), with mild EIAH classified as an SaO_2_ of 93%–95% (or >3% drop from baseline levels), moderate 88%–93%, and severe <88% (Dempsey & Wagner, 1999). EIAH occurs in approximately 50% of elite male athletes (V̇O_2_max >68 mL∙kg^−1^∙min^−1^) at maximal exercise but rarely in untrained males or males exercising at submaximal intensities (Powers et al., 1988). In contrast, about two‐thirds of females across varying fitness levels develop EIAH during both maximal and submaximal exercise (Dominelli et al., 2013; Harms et al., 1998; Richards et al., 2004). Despite these observations, research has not fully elucidated the mechanisms driving potential sex‐based differences in EIAH.

EIAH is thought to stem from one of four underlying mechanisms: (1) ventilation‐perfusion (V̇/Q̇) mismatch, (2) hypoventilation, (3) veno‐arterial shunt, and (4) diffusion limitation (Dempsey & Wagner, 1999). Diffusion limitation can result from the accumulation of fluid within the lungs, known as extravascular pulmonary edema. Extravascular pulmonary edema may develop during high‐intensity exercise when cardiac output (Q̇) is significantly elevated, which leads to high pressure on the peri‐alveolar vasculature (Hopkins et al., 1997; West et al., 1991). This condition is commonly termed exercise‐induced pulmonary edema. With a large surface area for gas exchange and minimal diffusion distance between the alveoli and pulmonary capillaries, the capillary blood vessels may be susceptible to leakage as a result of heightened pressures, and the subsequent buildup of extravascular fluid. In turn, there is an increase in the thickness of the alveolar‐capillary membrane, which may impede oxygen diffusion, and thus, arterial oxygen content. However, the question of whether exercise can induce pulmonary edema in humans remains controversial (Hopkins, 2010; Sheel & McKenzie, 2010).

In addition to previously mentioned sex differences in respiratory anatomy, differences in pulmonary vascular function may potentially contribute to an increased susceptibility of females to develop pulmonary edema during exercise. Sless et al. (2023) found elevated arterial elastance and higher total pulmonary resistance, alongside lower systemic and pulmonary compliance in healthy females compared to males. These results suggest an elevated vascular load in both systemic and pulmonary circulations in females. Further substantiating this concept, Lau et al. (2022) observed significant increases in arterial stiffness in females compared to males, albeit in individuals with heart failure with preserved ejection fraction. This heightened stiffness was associated with a notable rise in pulmonary capillary wedge pressure during exercise in females. These observations regarding sex‐specific pulmonary vascular dynamics emphasize the necessity for a more comprehensive understanding of how these factors may contribute to potential sex differences in exercise‐induced pulmonary edema development.

Many studies have explored non‐invasive imaging techniques, including chest radiography, magnetic resonance imaging (MRI), and computerized tomography (CT) to evaluate post‐exercise pulmonary edema with mixed results (Anholm et al., 1999; Caillaud et al., 1995; MacNutt et al., 2007; McKenzie et al., 2005; Zavorsky, Saul, Decker, & Ruiz, 2006). One critique of imaging studies is that there is often a relatively long delay between completion of exercise and image acquisition, which may allow sufficient time for the lymphatic system to clear excess fluid before the scans are obtained (Hopkins, 2010). Schaffartzik et al. (1992), suggested that gas exchange abnormalities and EVLW accumulation can resolve as quickly as 20 min following exercise. However, several human studies have reported persistent increases in lung density with CT (Caillaud et al., 1995) and MRI (McKenzie et al., 2005), over an hour following exercise cessation.

Lung ultrasound is an alternative and relatively inexpensive method for evaluating pulmonary edema that allows for rapid assessment following exercise in the laboratory or in a field setting. Quantification of pulmonary edema using lung ultrasound is obtained through quantification of “b‐lines”, which are indicative of elevated levels of extravascular fluid in the lungs (Frassi et al., 2008; Pingitore et al., 2011). B‐lines are attributed to differences in impedance between the fluid‐filled septa and the air‐filled lungs, which produces observable reflections that can be differentiated by ultrasound (Picano et al., 2006). B‐lines are perpendicular to the pleural line of the lung, creating a “wedge‐shaped” appearance, widening as they advance away from the origin (Pingitore et al., 2011). Research has demonstrated a positive correlation between b‐lines and extravascular lung water (EVLW) (*r* = 0.42, *p* = 0.001), wedge pressure (*r* = 0.48, *p* = 0.01), and radiologic lung water (*r* = 0.60, *p* = 0.0001) (Agricola et al., 2005; Jambrik et al., 2004). A systematic review further supported these findings, indicating that lung ultrasound has 97% and 98% sensitivity and specificity, respectively, in the detection of acute pulmonary edema (Wang et al., 2018). Comparative studies of lung ultrasound, chest CT, and radiography in those with alveolar interstitial syndrome have also concluded that b‐lines allow for the detection of interstitial pulmonary edema (Agricola et al., 2005; Lichtenstein et al., 1997).

In combination with high‐intensity exercise, integration of portable ultrasound and pulse oximetry provides a unique opportunity to assess sex differences in exercise‐induced pulmonary edema and hypoxemia directly in the field. Accordingly, the primary objective of this study was to examine sex differences in the magnitude of exercise‐induced pulmonary edema development and its corresponding effects on EIAH in highly trained female and male endurance athletes in a real‐world setting. We hypothesized that pulmonary edema development following high‐intensity time trial exercise would be greater and occur more frequently in females than males, which would be associated with lower oxygen saturations in females.

## 2 METHODS

### 2.1 Study participants

Twenty highly trained individuals (10 males and 10 females) were recruited for this study (age range, 18–55 years). Participants were required to have normal spirometry (i.e., forced expiratory volume in 1 s (FEV_1_) to forced vital capacity (FVC) ratio >0.70 and FEV_1_ ≥80% predicted), a body mass index >18 and <30 kg/m^2^, an aerobic capacity (V̇O_2_peak) greater than 120% predicted, and have previously completed the Grouse Grind® trail in North Vancouver, Canada (~2.5 km, ~800 m elevation gain). Participants were excluded if they were a past or current smoker, had a history of cardiorespiratory disease, or had contraindications to exercise testing. This study received institutional ethics approval from The University of British Columbia and Providence Health Care Research Institute Ethics Board (H22‐01321) and adhered to the *Declaration of Helsinki*, except for registration in a database. All participants provided written informed consent prior to participating in the study.

#### 2.1.1 Experimental overview

Participants attended two testing visits. The first visit took place at the laboratory where they provided written informed consent followed by completion of medical history and questionnaires addressing medication usage and training status. Height and mass were measured (Seca 769; Seca, CA, USA) followed by pulmonary function testing including spirometry, maximal voluntary ventilation (MVV), and diffusion capacity of the lungs for carbon monoxide (DLCO). Lastly, prior to exercise testing, the participants underwent a lung ultrasound performed by a trained study investigator. All of the aforementioned variables were used to characterize the study population and ultrasound data served as the baseline for the second experimental visit. Participants were familiarized with the testing procedures before completing an incremental treadmill exercise test to volitional fatigue to determine their V̇O_2_peak. The lung ultrasound measurements and DLCO were repeated approximately 10–15 min and 15–20 min, respectively, following the completion of the treadmill exercise test.

The second visit took place at the Grouse Grind® trail in North Vancouver, British Columbia, which begins at an elevation 290 meters above sea level and ascends to 1090 m above sea level. Participants were fitted with a chest strap to monitor heart rate (HR) and were then instructed to perform a maximal time trial effort up the course. During the final ~100 m of the Grouse Grind®, participants were handed a portable finger pulse oximeter (Fingertip Pulse Oximeter C20, ChoiceMMed, Beijing, China) to place on their index finger while completing the remaining ~100 m of the time trial. This allowed for immediate assessment of SpO_2_ levels upon completion of exercise. Approximately 10–15 min after completion of the time trial, participants underwent the same lung ultrasound assessment as in visit 1.

### 2.2 Exercise protocol

#### 2.2.1 Visit 1: Incremental treadmill exercise test

The incremental exercise test was performed on a treadmill (Trackmaster Treadmills, Full Vision, Inc.; Newton, Kansas, United States) using a similar protocol described previously (Ciavaglia et al., 2014; Muscat et al., 2015). The exercise test started at 30 W (Watts) and increased by 30 W in a stepwise fashion every 2 min until volitional exhaustion. To achieve the desired work rate, participant body mass was inputted into a formula to determine the treadmill speed for each stage while the treadmill grade was standardized such that the test started at a 5% incline and then increased by 1% per stage (Jones, 1997). Standard cardiorespiratory measures were continuously recorded and averaged over 30‐s epochs using a commercially available cardiopulmonary testing system (TrueOne 2400; Parvo Medics, Salt Lake City, Utah, US). SpO_2_ was measured using finger pulse oximetry (Nonin 7500, Nonin Medical Inc., Plymouth, US). HR was measured using a commercially available HR monitor strap around participants' chest (Garmin Ltd., Olathe, Kansas, USA).

#### 2.2.2 Visit 2: Time trial on Grouse Grind®

Participants were met at the base of the Grouse Grind® in North Vancouver by a member of the research team. To measure HR during exercise, they were fitted with the same commercially available HR monitor used on visit 1. Participants were then instructed to complete the Grouse Grind® trail in the shortest amount of time possible. Time was recorded on a stopwatch (Model 226, Sportline, US) through communication with a study investigator who was waiting to meet participants at the top of the Grouse Grind®. Once the participant crossed the finish line, the time was stopped and recorded along with their SpO_2_. We aimed to complete the lung ultrasound measurements between 10‐ and 15‐min following completion of the time trial.

### 2.3 Measurements

#### 2.3.1 Pulmonary function testing (visit 1)

Spirometry, MVV, and DLCO were performed according to standard recommendations (Graham et al., 2017, 2019; Wanger et al., 2005) using a commercially available cardiopulmonary testing system (Vmax 229d with Autobox 6200 DL; SensorMedics, Yorba Linda, CA, USA). Data were expressed as absolute values and as percent predicted (American Thoracic Society, 1995; Cooper et al., 2017; Gutierrez et al., 2004).

#### 2.3.2 Lung ultrasound b‐lines (visits 1 and 2)

Lung ultrasound was performed before and after exercise on visit 1 and after exercise on visit 2 by a researcher trained in the technique, according to an established protocol (Volpicelli et al., 2006). Ultrasound assessment was performed with participants in the supine position. An 8‐zone examination of the lungs was performed using a phased array probe and a scanning depth set to approximately 16 cm (Jambrik et al., 2004) (Lumify S4‐1 Phased Array Transducer, Phillips, Amsterdam, Netherlands).

**FIGURE 1:**
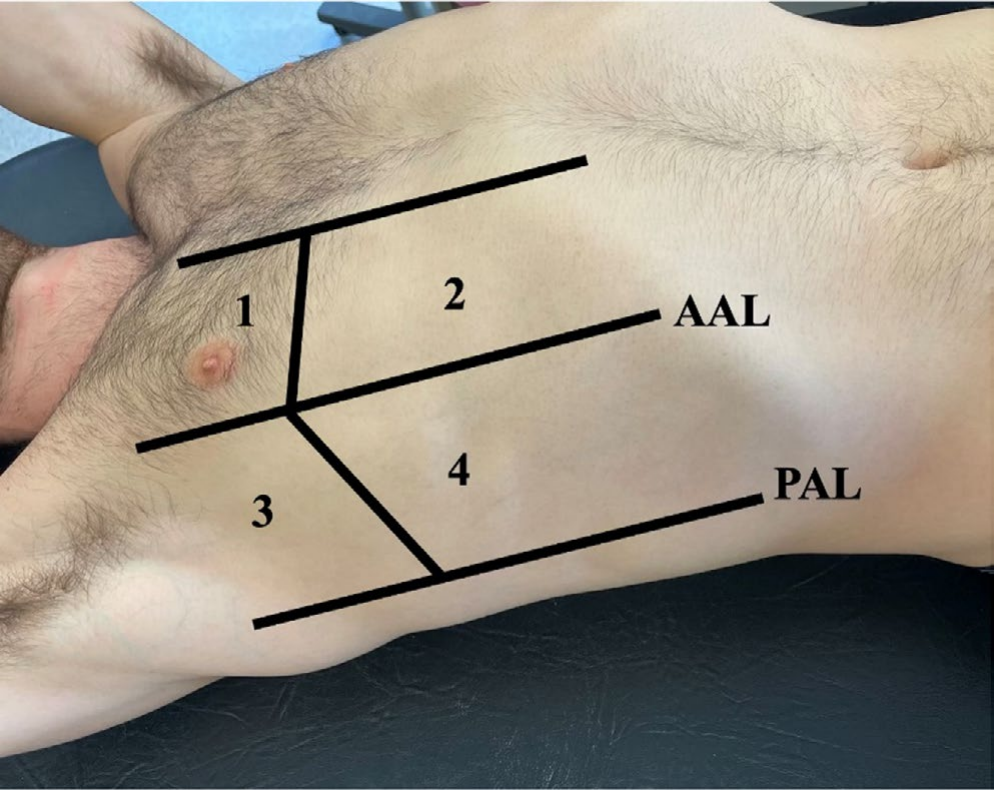
8‐Zone lung ultrasound examination of a representative participant. Areas 1 and 2 denote the anterior chest regions. Areas 3 and 4 denote the lateral chest regions. The regions were the same on the left side of the chest region as seen above (Volpicelli et al., 2006).

The chest was divided into two hemithoraces. Each hemithorax contained four assessment zones: two anterior and two lateral. The anterior zones were delineated by the sternal border and the anterior axillary lines. The lateral zone was delineated by the anterior and posterior axillary lines. Both anterior and lateral zones were further divided into cephalad and caudad segments, delineated by the fourth rib (nipple level) (see Figure 1). Within the intercostal spaces of each zone, the number of b‐lines was recorded, and added to create a “b‐line score”, which was subsequently summed across all eight zones. A score of 0–2 b‐lines indicated no pulmonary edema development, 2–4 was mild edema, 5–9 was moderate edema, and ≥10 b‐lines was indicative of severe edema (Picano et al., 2006). The scoring of b‐lines was assessed by two independent study investigators who had received standardized training, and were blinded to both timing of the scan, as well as the participant identification.

### 2.4 Statistical analysis

A Shapiro–Wilk test was used to test the normality of the data. Sex differences in descriptive characteristics were assessed using the Mann–Whitney *U*‐test. The Wilcoxon signed‐rank test was used to compare pre‐ and post‐exercise b‐line scores on both visits and between sexes. The Cohen's Kappa coefficient was used to evaluate the interrater agreement between the two ultrasound assessors. To determine sex differences in EIAH, SpO_2_ values were taken at peak exercise on both visits and comparisons between sexes were made using a Freidman analysis, where sex was the between‐subject factor and visit was the within‐subject factor. Pearson correlation coefficients were used to determine the relationship between SpO_2_ values and b‐line scores on both visits. All data are presented as median (interquartile range (IQR)). Statistical testing was performed on SPSS software (v28.0, SPSS Inc., Chicago, IL, USA). In all cases, a *p* < 0.05 was considered statistically significant.

## 3 RESULTS

### 3.1 Participant characteristics

Twenty individuals (*n* = 10 males, *n* = 10 females) participated in this study, although one female did not complete visit 2 because the study team was unable to schedule her visit before the seasonal closure of the Grouse Grind® trail. Average self‐reported personal best times on the Grouse Grind® trail were 29.2 (9.3) min for males and 37.5 (5.0) min for females. Participant characteristics are presented in Table 1. Females and males were well‐matched for age and body mass index, although males, on average, were taller and had a greater FVC and FEV_1_ compared to females.

**TABLE 1:** Participant characteristics.

	Males (*n* = 10)	Females (*n* = 10)
*Participants*		
Age, years	31.0 (12.0)	37.5 (14.3)
Height, cm	176.0 (5.8)	169.5 (7.8)*
Mass, kg	67.5 (8.5)	60.7 (5.0)*
Body mass index, kg∙m^−2^	21.6 (2.0)	21.4 (2.9)
Moderate PA, min∙week^−1^	417 (204)	219 (152)*
Vigorous PA, min∙week^−1^	374 (323)	372 (387)
*Spirometry*		
FVC, L	5.41 (0.78)	4.08 (1.14)*
FVC, % predicted	101 (10)	101 (16)
FEV_1_, L	4.29 (0.26)	3.41 (0.59)*
FEV_1_, % predicted	102 (10)	102 (10)
FEV_1_/FVC, %	78.5 (12.3)	81.0 (4.8)
MVV, L∙min^‐1^	195 (29)	131 (24)*

*Note*: Values are medians (Interquartile range (IQR)).

Abbreviations: FEV_1_, forced expiratory volume in 1 s; FVC, forced vital capacity; MVV, maximum voluntary ventilation; PA, physical activity.

* *p* < 0.05 statistically different between sexes.

### 3.2 Cardiorespiratory, metabolic, and respiratory responses to incremental treadmill exercise (visit 1)

The cardiorespiratory responses to peak treadmill exercise from visit 1 are displayed in Table 2. Males achieved higher absolute and relative V̇O_2_peak values and exercised for longer compared to females, but both groups were well‐matched for % predicted V̇O_2_peak according to Jones et al. (1985) [M: 161 (17) vs. F: 156 (36) % predicted, respectively, *p* = 0.94]. There was no correlation between b‐lines and V̇O_2_max (*r* = 0.12, *p* = 0.60). SpO_2_ at peak exercise was not significantly different between males and females (*p =* 0.76). In addition, there was no significant sex difference in the change in DLCO pre‐ versus post‐exercise [M: −4.6 (9.4) vs. F: −3.9 (14.5) % change, *p =* 0.59], which was measured at 18.0 (2.0) min following exercise cessation.

**TABLE 2:** Peak responses to exercise between sexes on visit 1 incremental treadmill test.

Peak incremental data	Males (*n* = 10)	Females (*n* = 10)
Exercise duration, min	20.2 (2.5)	12.6 (2.0)*
HR, beats∙min^−1^	186 (8)	182 (10)
HR, % predicted	97 (2)	97 (7)
V̇O_2_, mL∙kg^−1^∙min^−1^	69.3 (8.8)	50.7 (4.1)*
V̇O_2_, % predicted	161 (17)	156 (36)
V̇O_2_, L∙min^−1^	4.84 (0.41)	3.15 (0.41)*
V̇CO_2_, L∙min^−1^	5.34 (0.45)	3.48 (0.59)*
RER	1.09 (0.06)	1.09 (0.02)
V̇_E_, L∙min^−1^	153 (9)	100 (27)*
V̇_E_/MVV, %	79.5 (12.4)	78.9 (18.7)
V_T_, L	2.65 (0.58)	2.15 (0.50)*
F_b_, breaths∙min^−1^	54 (8)	48 (4)
V̇_E_/V̇O_2_	31 (2)	32 (6)
V̇_E_/V̇CO_2_	29 (1)	29 (4)

*Note*: Values are median (IQR).

Abbreviations: F_b_, breathing frequency; HR, heart rate; MVV, maximum voluntary ventilation; RER, respiratory exchange ratio; V̇CO_2_, rate of carbon dioxide production; V̇_E_, minute ventilation; V̇O_2_, rate of oxygen consumption; V_T_, tidal volume.

* *p* < 0.05 statistically different between sexes.

### 3.3 Peripheral oxygen saturation (SpO_2_ ) and EIAH (visit 1)

**FIGURE 2:**
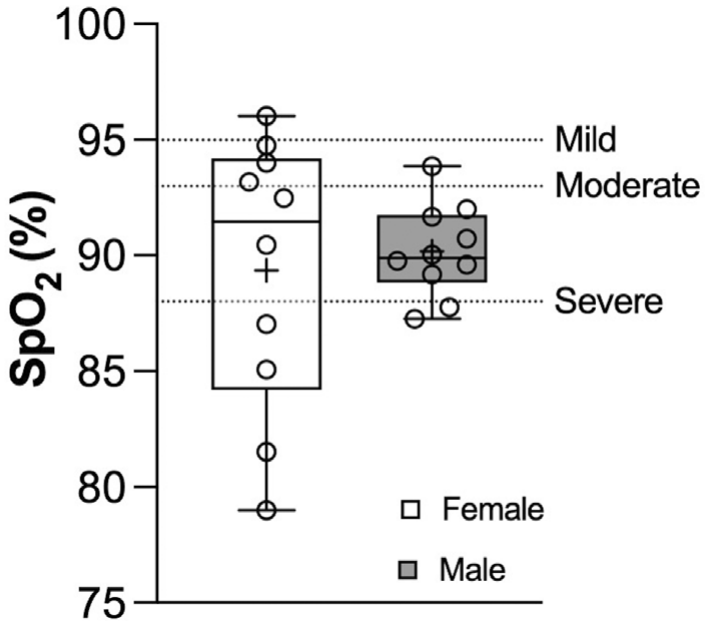
Sex differences in SpO_2_ at peak exercise on visit 1. The box region is the IQR, the horizontal line is the median, and the lines outside of the box represent the minimum and maximum values. The dots represent individual data points. +, mean value of all respective participants. The horizontal dashed lines represent the severity classification of EIAH based on SaO_2_ (Dempsey & Wagner, 1999).

There was no significant sex difference in SpO_2_ between males and females at peak exercise on the treadmill test (Figure 2) [M: 90 (2) vs. F: 91 (8) %, *p* = 0.76]. There was a 9 (3) and 6 (8) percentage point decrease in SpO_2_ from baseline to peak exercise in males and females, respectively. According to the classifications of EIAH (Dempsey & Wagner, 1999), albeit for SaO_2_ instead of SpO_2_, two of the male participants developed severe EIAH, seven moderate, and one mild, whereas four females developed severe EIAH, two moderate, and three mild EIAH during incremental exercise.

### 3.4 Ultrasound b‐lines (visit 1)

**FIGURE 3:**
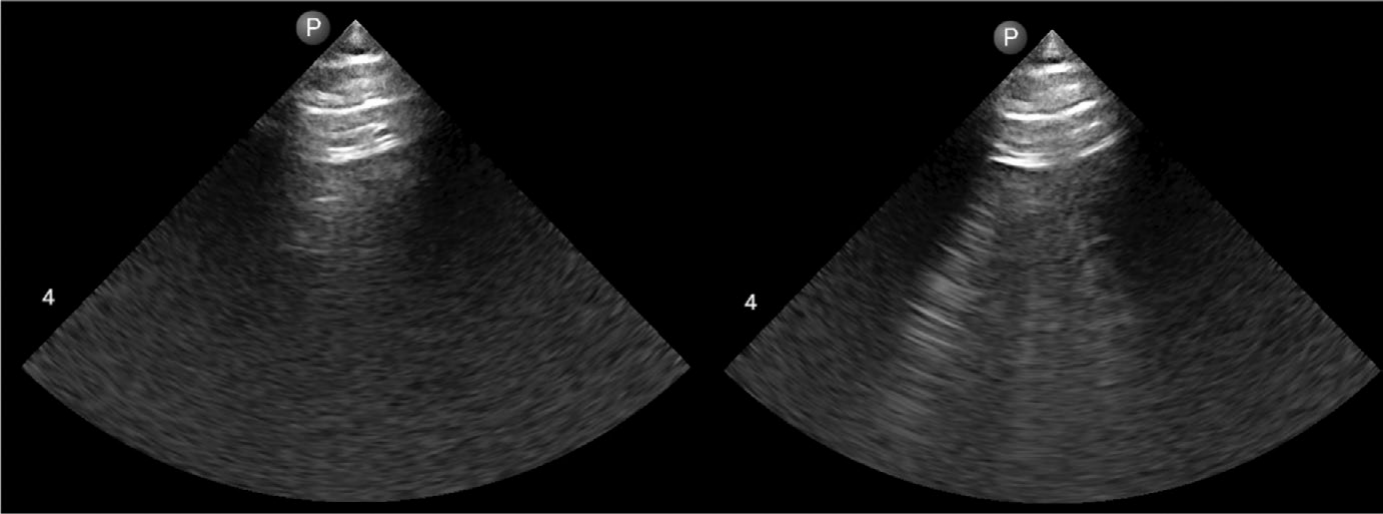
Lung ultrasound in a single participant on visit 1, pre (left) and post (right) exercise that developed mild pulmonary edema.

Two raters were responsible for scoring the b‐lines. The interrater agreement on the lung ultrasound scans was 94.7%, with a Cohen's Kappa coefficient of 0.64. There were minimal b‐lines present at baseline, with no differences between males and females [M: 0.8 (0.9) vs. F: 0.5 (1.3), *p* = 0.76]. Similarly, following exercise there were no differences between males and females in b‐line scores [M: 1.0 (0.9) vs. F: 1.0 (1.9), *p* = 0.76]. Additionally, there was no significant correlation between peak exercise SpO_2_ and the presence of b‐lines when males and females were pooled together (*r* = 0.236, *p* = 0.32). Lung ultrasound in a single participant pre‐ and post‐exercise is displayed in Figure 3. Average time‐to‐scan post‐exercise completion on visit 1 was 13.1 (1.5) min in males and 13.5 (1.5) in females.

### 3.5 EIAH and ultrasound b‐lines during Grouse Grind® time trial (visit 2)

**FIGURE 4:**
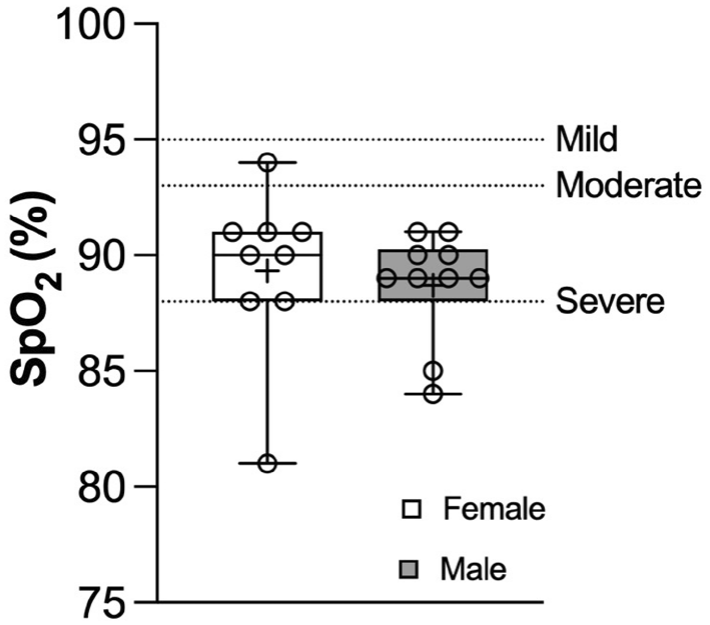
Sex differences in SpO_2_ at peak exercise on visit 2. The box region is the IQR, the horizontal line is the median, and the lines outside of the box represent the minimum and maximum values. The dots represent individual data points. +, mean value of all respective participants. The horizontal dashed lines represent the severity classification of EIAH based on SaO_2_ (Dempsey & Wagner, 1999).

**FIGURE 5:**
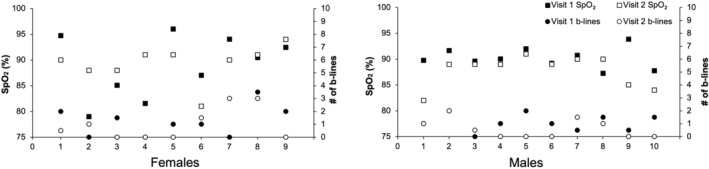
Individual SpO_2_ levels and ultrasound b‐lines across both study visits. Females are displayed on the left and males on the right. The *x*‐axis represents individual participants, while the *y*‐axis shows SpO_2_ (%) measured at peak exercise on both visits, and the right *y*‐axis shows the number of b‐lines observed on ultrasound scans taken post‐exercise during both visits. Solid markers indicate data from visit 1 and open markers indicate data from visit 2.

Testing on the Grouse Grind® was done in late September and early October. The average temperature and relative humidity on testing days was 15.6°C and 91.7%, respectively, which was higher than the typical humidity for that time of year. Time trial duration was significantly shorter in males relative to females (M: 31.5 (6.5) vs. F: 40.4 (7.5) min, *p* = 0.006). Females sustained a higher average percentage of the maximum incremental treadmill HR for the duration of the time trial (M: 94 (1) vs. F: 96 (1) %max, *p* = 0.025). However, males and females sustained similar average absolute HR throughout exercise (M: 170 (8) vs. F: 174 (2) bpm, *p* = 0.24). At peak exercise on visit 2, HR was not significantly different between sexes whether expressed as a percentage of maximum incremental treadmill HR [M: 98 (2) vs. F: 99 (1) %max, *p* = 0.18] or absolute HR [M: 178 (1) vs. F: 179 (1) bpm, *p* = 0.51]. SpO_2_ was also not significantly different between males and females (Figure 4) [M: 89 (4) vs. F: 90 (3) %, *p* = 0.41]. Two males developed severe EIAH and eight moderate EIAH at peak exercise. Of the female participants, one developed severe EIAH, seven moderate EIAH, and one mild EIAH. There were no differences between male and female b‐line scores following time trial exercise [M: 0.3 (1.0) vs. F: 0.5 (1.5), *p* = 0.60]. Within‐subjects SpO_2_ and b‐lines across both visits can be seen in Figure 5. Similar to visit 1, the Pearson correlation coefficient between SpO_2_ and b‐lines was not significant when all the participants were pooled together (*r* = 0.052, *p* = 0.84).

## 4 DISCUSSION

### 4.1 Primary findings

The primary aim of this study was to investigate potential sex differences in exercise‐induced pulmonary edema and EIAH development in highly trained athletes following near‐maximal exercise in a real‐world setting. Our findings are two‐fold: (1) following both incremental treadmill and time trial exercise, there was minimal development of pulmonary edema in both males and females, as assessed by lung ultrasound and (2) there was no difference in the number of males and females who developed EIAH or the magnitude of desaturation during both incremental treadmill exercise and time trial exercise in the field. These findings suggest that highly trained male and female athletes appear to experience a similar magnitude of EIAH development that cannot be explained by the development of pulmonary edema during both laboratory and field‐based exercise testing.

### 4.2 Pulmonary edema

We reasoned that the combination of smaller lungs, narrower airways and a reduced overall surface area for pulmonary gas exchange (Dominelli et al., 2018; Mead, 1980), coupled with potentially high pulmonary vascular load, pulmonary vascular resistance and arterial stiffness during exercise (Lau et al., 2022; Sless et al., 2023), as well as a potentially greater susceptibility to EIAH (Dominelli et al., 2013; Harms et al., 1998), may increase the likelihood of females developing exercise‐induced pulmonary edema compared to males. Contrary to our hypothesis, we observed no discernable differences between males and females in the magnitude and frequency of pulmonary edema development during both laboratory‐based cardiopulmonary exercise testing and field‐based time trial exercise. The lack of significant increase in b‐line score from baseline to post‐exercise in both sexes aligns with previous work in males and females across separate studies that showed no evidence of pulmonary edema on chest CT after intense hypoxic cycling exercise (Guenette et al., 2007; MacNutt et al., 2007). However, our findings are contrary to previous work by Zavorsky, Saul, Murias, and Ruiz (2006) and McKenzie et al. (2005) that identified evidence of mild pulmonary edema in trained female and male cyclists following high‐intensity exercise. As mentioned previously, research examining pulmonary edema development following exercise has produced conflicting results (Guenette et al., 2007; Hodges et al., 2007; MacNutt et al., 2007; Pingitore et al., 2011; Zavorsky, Saul, Decker, & Ruiz, 2006), leading to debate in the literature (Hopkins, 2010; Sheel & McKenzie, 2010). Much debate surrounds the imaging modality and methodologies used to assess pulmonary edema development, such as CT, MRI, and Bronchoalveolar Lavage (BAL), with no clear consensus (Boussuges & Gargne, 2010; Hopkins, 2010; Sheel & McKenzie, 2010; Zavorsky & Anholm, 2010). In addition, researchers have questioned whether the findings of pulmonary edema are a reflection of underlying undiagnosed pathologies in the participants or a direct result of exercise, given that many of the cases reflect a small number of individuals as opposed to a larger sample of participants within an exercise trial (Eldridge, 2010; Sheel & McKenzie, 2010). The debate will likely persist until a standardized assessment model with a larger sample of individuals is obtained.

Considering the absence of pulmonary edema development in either sex, especially among highly trained endurance athletes who consistently push the respiratory system to its limits, it may become reasonable to explore alternative physiological factors that may contribute to the development of pulmonary edema (Dempsey et al., 2020; Ekblom & Hermansen, 1968). Given that hydrostatic pulmonary edema primarily arises from elevated pulmonary vasculature pressure (Staub, 1978), it appears reasonable to speculate that HR, and subsequent Q̇ during exercise, may function as potential predictors of pulmonary edema development. In a review from Zavorsky (2007), it was found that time trial effort was four times more likely to elicit pulmonary edema than maximal incremental or submaximal exercise testing. This was attributed to the sustained higher Q̇ over prolonged durations, implying that a combination of exercise intensity and duration may contribute to pulmonary edema development. During our second visit, where participants completed the Grouse Grind® time trial, the average HR sustained was 94 (1) % of peak obtained on visit 1 in males and 96 (1) % in females, over an average duration exceeding 30 min. According to the aforementioned rationale, one may expect that if pulmonary edema development were to occur, it would have been during this time trial. Surprisingly, we observed no evidence of pulmonary edema development during the sustained time trial efforts. These observations may suggest that exercise intensity and duration may not be the key underlying mechanisms of pulmonary edema development.

Considering elite athletes are capable of elevating their Q̇ five to eight times above resting values (Ekblom & Hermansen, 1968), it has been postulated that highly trained individuals may approach the limits of their respiratory system. The elevated Q̇ results in a substantial rise in pulmonary capillary blood volumes and subsequent pressures during exercise (Ekblom & Hermansen, 1968; Harms et al., 1998). In a systematic review of pulmonary pressures during exercise, Kovacs et al. (2009) observed that increases in pulmonary arterial pressure during exercise occur as a function of increasing Q̇, which potentially alludes to higher pressures achieved among trained individuals. Additionally, they found the upper limit of normal pulmonary arterial pressures during maximal exercise to be 36.8 mmHg within a healthy population, which is intriguing when considered alongside the findings of West et al. (1991), who demonstrated that capillary transmural pressures of 40 mmHg in rabbits were sufficient to induce damage to the capillary endothelium. Expanding upon this, West (2004) found intense exercise can elevate pulmonary artery wedge pressures to as high as 37 mmHg, leaving minimal reserve for the pulmonary capillaries to maintain their structural integrity. Therefore, considering this rationale, individuals with higher absolute V̇O_2_max values, and Q̇, may be more vulnerable to respiratory system limitations, such as exercise‐induced pulmonary edema. However, our observations did not reveal any correlation between EVLW development, as indicated by b‐line scores, and V̇O_2_max. Notably, Harms et al. (1998) did observe a correlation between end‐exercise SaO_2_ and V̇O_2_max among females, which alludes to the potential relationship between fitness level and EIAH development. Future studies across a wider range of fitness levels are needed to further examine the relationship between aerobic fitness, EVLW and EIAH. Additionally, further investigation into disruptions of the alveolar‐capillary membrane and their potential implications on gas exchange during maximal exercise is necessary.

### 4.3 EIAH and pulmonary edema

The development of EIAH during exercise suggests impairments in oxygen delivery to the locomotor muscles (Dempsey & Wagner, 1999). On average, our participants exhibited an eight‐percentage point decrease in SpO_2_ relative to baseline levels. In accordance with the SaO_2_ criteria outlined by Dempsey and Wagner (1999), many of our participants developed EIAH of varying severity, albeit using SpO_2_ instead of SaO_2_, with no discernable differences between males and females. These findings alongside the absence of pulmonary edema detected in our athletes, highlight a dissociation between EVLW development and impaired gas exchange, as discussed by MacNutt et al. (2007). Interestingly, this is contrary to work by Zavorsky, Saul, Murias, and Ruiz (2006), who demonstrated mild pulmonary edema in 14 females following repeated bouts of intense cycle exercise, without a corresponding impairment in gas exchange (Zavorsky, Saul, Decker, & Ruiz, 2006). While seemingly contradictory, these results collectively suggest alternative factors contributing to EIAH development, in addition to the potential sex‐based differences in EIAH previously reported (Dominelli et al., 2013; Richards et al., 2004).

While direct examination of sex differences in EIAH is lacking, comprehensive reviews on sex and gender differences on pulmonary physiology (Dominelli & Molgat‐Seon, 2022; Hopkins & Harms, 2004) note a higher proportion of females (~65%) compared to males (~50%) develop EIAH. Moreover, Hopkins and Harms (2004) highlight that almost 12% of females with a V̇O_2_ less than 50 mL∙kg^−1^∙min^−1^ (across multiple studies) develop EIAH, compared to only 2% of males with similar fitness levels. The reasons for these sex‐based differences are still unclear. There is reason to believe that this is due, at least in part, to ventilatory constraints, since EIAH can be partially reversed when ventilatory constraints are attenuated with heliox breathing in females (Dominelli et al., 2013) and males (Dempsey et al., 1984). Anatomical differences, such as smaller lung volumes and lower maximal flow rates, may make females more susceptible to ventilatory limitations and, consequently, EIAH development. Interestingly, our results did not show sex difference in EIAH development. The median peak exercise SpO_2_ values on visit 1 were 90% in males and 91% in females, with similar values during time trial exercise on visit 2 (89% in males and 90% in females). Thus, our findings do not support the notion that females are more susceptible to pulmonary gas exchange abnormalities than males, at least in our relatively small group of high‐performance endurance athletes using non‐invasive pulse oximetry. As acknowledged in a review from Hopkins and Harms (2004), further research needs to be done with arterial blood gas analysis to elucidate mechanisms that underlie potential sex differences in EIAH.

### 4.4 Limitations

We recognize that there are several limitations of the present study. First is the use of a pulse oximeter to indirectly measure SaO_2_. Although pulse oximetry has been shown to be a valid measure of blood oxygen saturation in elite athletes (Martin et al., 1992), we acknowledge that arterial blood gas measures are more precise. However, logistical constraints and our desire to minimize invasiveness (to optimize participant recruitment) prevented the measurement of temperature‐corrected arterial blood gases. In addition, the use of different pulse oximeter devices between visits introduces potential error when making direct comparisons between the field and laboratory‐based exercise tests, as the measurement accuracy and algorithms may vary between devices. Second, due to logistical challenges associated with testing during the Grouse Grind® visit, we used visit one baseline ultrasound data as the baseline for visit two. However, many, if not all, participants completed visit two less than 2 weeks after visit one. Therefore, baseline measures from visit one should be reasonably similar for both visits. Third, as with all field‐based exercise studies, uncontrollable environmental conditions (e.g., air temperature, humidity, air quality, etc.) or trail congestion with other users, may have impacted some of our results. However, participant feedback following the time trial suggested minimal influence of the environmental and trail conditions on their performance. Additionally, we acknowledge our findings may not be generalizable to other exercise settings, such as altitude and swimming where environmental and physiological factors, such as immersion effects, swimming induced pulmonary edema, or hypoxia, may uniquely contribute to the development of EIAH. Furthermore, we acknowledge that lung ultrasound is limited by its subjective interpretation, reliance on a trained operator, and its ability to only capture sections of the lungs (Jozwiak et al., 2015). While the 8‐zone examination method used by other researchers should ensure adequate coverage of the lungs (Volpicelli et al., 2006), larger studies are needed to confirm the effectiveness of b‐lines to measure EVLW, as highlighted in a recent review by Parks et al. (2023).

### 4.5 Conclusion and future directions

Despite our efforts to optimize the conditions for pulmonary edema development by recruiting highly trained athletes and subjecting them to sustained, near‐maximal exercise in a real‐world setting, our results did not reveal evidence of pulmonary edema in both males and females. This suggests that the pulmonary vasculature has the structural integrity required to withstand the elevated cardiac outputs of highly trained athletes during intense exercise. Furthermore, a notable number of participants experienced EIAH, with no differences between sexes, indicating that EIAH is likely driven by mechanisms independent of pulmonary edema. Additional studies are needed to explore potential sex differences in the development and underlying mechanisms of both EIAH and pulmonary edema.

## AUTHOR CONTRIBUTIONS

JG, MK, and AH conceived and designed the research experiment. AH, RM, ON, and MF collected the data. AH, RM, and SS analyzed the data. AH, RM, KM, MK and JG interpreted the results of the data. AH drafted the manuscript and prepared the figures. JG, SS, RM, KB, KM, and MK edited and revised the manuscript. All authors approved the final version of the manuscript.

## FUNDING INFORMATION

This work was funded by a Discovery Grant from the Natural Sciences and Engineering Research Council (NSERC) of Canada. AH was funded 4‐year Fellowship (4YF) from The University of British Columbia and a Respiratory Rehabilitation Fellowship from the British Columbia Lung Foundation. ONF was supported by a 4YF, a Respiratory Rehabilitation Fellowship from the British Columbia Lung Foundation, a studentship from the Canadian Institutes of Health Research and Canadian Lung Association, and a CANTRAIN‐CTTP & Michael Smith Health Research BC Doctoral Studentship Award. KM received postdoctoral fellowship support from Michael Smith Health Research British Columbia and the Academic Enhancement Fund from the Division of Respiratory Medicine at The University of British Columbia.

## CONFLICT OF INTEREST STATEMENT

All authors declared they have no competing interests as a result of the e publication of this manuscript.

## ETHICS STATEMENT

This study recieved institutional ethics approval from The University of British Columbia and Providence Health Care Research Institute Ethics Board (H22‐01321). All study procedures adhered to the declaration of Helsinki, except for registration in a database. Informed consent was obtained from all individuals who participated in the study.

## Data Availability

Data is available for the above study upon reasonable request and following appropriate ethical approval and data transfer agreements.
